# The relationship between foot type and lower limb kinematics during walking: a systematic review

**DOI:** 10.1186/1757-1146-5-S1-P4

**Published:** 2012-04-10

**Authors:** Andrew K Buldt, George S Murley, Paul Butterworth, Pazit Levinger, Hylton B Menz, Karl B Landorf

**Affiliations:** 1 Department of Podiatry, Faculty of Health Sciences, La Trobe University, Bundoora 3086, VIC, Australia; 2 Musculoskeletal Research Centre, Faculty of Health Sciences, La Trobe University, Bundoora 3086, VIC, Australia

## Background

Variations of foot type, such as a low- or high-arched foot, are thought to be an intrinsic risk factor for lower extremity injury. One of the proposed mechanisms by which foot posture may contribute to injury is via altered motion of the lower extremity. Therefore, the aim of this review was to investigate the relationship between foot type and dynamic lower limb and foot kinematics during walking.

## Materials and methods

A systematic database search of MEDLINE, CINAHL, SPORTDiscus, Embase and Inspec was undertaken in May 2011. Two independent reviewers applied predetermined inclusion criteria to select articles for review. A two stage quality assessment was also undertaken for selected articles. Firstly, methodological quality was assessed using a modified version of the Quality Index. Secondly, kinematic methodology and reporting were assessed using a series of items derived from relevant references.

## Results

A final selection of 14 articles was reviewed from an initial list of 3470 citations. Meta-analysis was not conducted due to heterogeneity between studies. Quality Index scores ranged from 44-94% (mean 74%) and kinematic methodology, while varied, was generally repeatable. Selected articles mainly focused on low-arched and normal foot types. Articles could be grouped into two broad categories. Firstly, studies that compared mean differences found some evidence for increased motion in low-arched feet, but this was limited by small effect sizes. Secondly, studies that investigated associations found that a more pronated foot type was correlated with increased peak rearfoot eversion and total rearfoot eversion range of motion during walking.

## Conclusions

There is some evidence for a relationship between low-arched feet and increased motion during gait, however this was not conclusive due to heterogeneity between studies and small effect sizes. Future research should focus on a broader range of foot types including high-arched feet.

